# Mitochondrial Health in Aging and Age-Related Metabolic Disease

**DOI:** 10.1155/2016/5831538

**Published:** 2016-07-13

**Authors:** David Sebastián, Rebeca Acín-Pérez, Katsutaro Morino

**Affiliations:** ^1^Institute for Research in Biomedicine (IRB Barcelona), The Barcelona Institute of Science and Technology, Baldiri Reixac 10-12, 08028 Barcelona, Spain; ^2^Departament de Bioquímica i Biomedicina Molecular, Facultat de Biologia, Universitat de Barcelona, 08028 Barcelona, Spain; ^3^Instituto de Salud Carlos III, Centro de Investigación Biomédica en Red de Diabetes y Enfermedades Metabólicas Asociadas (CIBERDEM), Madrid, Spain; ^4^Centro Nacional de Investigaciones Cardiovasculares (CNIC), 28029 Madrid, Spain; ^5^Division of Endocrinology and Metabolism, Department of Medicine, Shiga University of Medical Science, Tsukinowa, Seta, Otsu, Shiga 520-2192, Japan

Aging is a biological process characterized by a progressive accumulation of damage that finally leads to disrupted metabolic homeostasis leading to cell damage. One of the hallmarks of aging is the presence of mitochondrial dysfunction translated in a decline in mitochondrial respiration and oxidative capacity, reduced mitochondrial mass, morphological alterations of mitochondria, and increase in oxidative stress. Therefore, preservation of mitochondrial health is crucial for maintaining tissue homeostasis during aging. Several mitochondrial quality control mechanisms have been identified, such as pathways related to protein folding and degradation, also known as mitochondrial unfolding protein response (mtUPR), as well as pathways involved in the turnover of mitochondria (mitophagy). However the direct link between quality control mechanisms and loss of mitochondrial health observed during aging and age-related alterations is still unknown. In this special issue, different research articles, performed by independent groups, address the role of mitochondrial function in the frame of aging or age-related pathologies using different experimental approaches and models. Although mitochondrial fitness is declined due to different pathological alterations, most of them converge in two critical hotspots: increase of reactive oxygen species leading to oxidative damage and metabolic maladaptation as leading causes of unhealthy aging.

H. Yamamoto et al. investigated the effects of a traditional Indian medicinal plant, Amla, on mitochondrial function. Amla has been shown to improve age-related pathology by decreasing oxidative stress. In their article, H. Yamamoto et al. show that Amla increases mitochondrial function and biogenesis, together with an activation of Nrf2, which together explain the antioxidant actions of Amla.

In their research article, A. V. Lokhmatikov et al. study the capacity of several natural antioxidants in the maintenance of mitochondrial health. Mitochondrial cardiolipin (CL) oxidation is increased in aging and age-related pathologies and is considered one of the first signaling elements of the mitochondrial apoptotic pathway. By using a chemical model resembling the mitochondrial membrane, they show that amphiphilic antioxidants can prevent oxidation of CL trapped within the respiratory supercomplexes and then preserve mitochondrial function.

Mitochondrial dysfunction has been also associated with coronary heart disease (CHD) and diabetes. O. M. Duicu et al. have found a more severe mitochondrial dysfunction in patients with CHD and diabetes than in patients with only CHD, characterized by a lower mitochondrial respiration. Importantly, this mitochondrial dysfunction is associated with increase in oxidative stress which was reduced when monoamine oxidase (MAO) was inhibited, pointing out to MAO as an important contributor to oxidative stress in CHD.

Neurodegeneration is also associated with aging and mitochondrial dysfunction. In this special issue, two research articles address the role of mitochondria in neurodegenerative diseases. F. Zhao et al. study the role of AP39, a new hydrogen sulphide donor with beneficial effects in Alzheimer's disease (AD). They found that AP39 sustains mitochondrial bioenergetics in neurons coming from an AD mouse model, leading to a decrease in ROS and an increase in cell viability. Moreover, administration of this compound to a mouse model of AD inhibited brain atrophy and ameliorated the alterations seen in AD. K. Peng et al. address the protective effect of resveratrol on a rotenone-induced model of Parkinson's disease (PD). They demonstrate that resveratrol increases mitochondrial mass and fusion and decreases ROS levels, leading to neurotoxicity protection, increased survival rate, and motor ability.

Finally, R. Pääsuke et al. evaluate the alterations in energy metabolism in muscle satellite cells during aging. By using myoblasts isolated from biopsies from young and old subjects, they show that both aging* in vitro* (increase in passages) and aging* in vivo* (myoblasts from young and old subjects) are associated with a reduced mitochondrial respiration and increased glycolysis.

Taken altogether, the data presented in the different research articles of this special issue suggest that manipulations aimed at preserving mitochondrial function and health, mainly targeting oxidative damage and metabolic adaptations, could contribute to the amelioration of aging and age-related pathologies and could help in the development of novel therapeutic approaches.

*David Sebastián* *David Sebastián* *Rebeca Acín-Pérez* *Rebeca Acín-Pérez* *Katsutaro Morino* *Katsutaro Morino*

